# A Tea Buds Counting Method Based on YOLOv5 and Kalman Filter Tracking Algorithm

**DOI:** 10.34133/plantphenomics.0030

**Published:** 2023-03-30

**Authors:** Yang Li, Rong Ma, Rentian Zhang, Yifan Cheng, Chunwang Dong

**Affiliations:** ^1^ Key Laboratory of Tea Quality and Safety Control, Ministry of Agriculture and Rural Affairs, Tea Research Institute, Chinese Academy of Agricultural Sciences, Hangzhou, China.; ^2^ College of Optical, Mechanical and Electrical Engineering, Zhejiang A&F University, Hangzhou, China.; ^3^ Tea Research Institute of Shandong Academy of Agricultural Sciences, Jinan 250100, China.; ^4^ College of Mechanical and Electrical Engineering, Shihezi University, Shihezi, China.

## Abstract

The tea yield estimation provides information support for the harvest time and amount and serves as a decision-making basis for farmer management and picking. However, the manual counting of tea buds is troublesome and inefficient. To improve the efficiency of tea yield estimation, this study presents a deep-learning-based approach for efficiently estimating tea yield by counting tea buds in the field using an enhanced YOLOv5 model with the Squeeze and Excitation Network. This method combines the Hungarian matching and Kalman filtering algorithms to achieve accurate and reliable tea bud counting. The effectiveness of the proposed model was demonstrated by its mean average precision of 91.88% on the test dataset, indicating that it is highly accurate at detecting tea buds. The model application to the tea bud counting trials reveals that the counting results from test videos are highly correlated with the manual counting results (*R*^2^ = 0.98), indicating that the counting method has high accuracy and effectiveness. In conclusion, the proposed method can realize tea bud detection and counting in natural light and provides data and technical support for rapid tea bud acquisition.

## Introduction

For many people, tea is one of the most important drinks taken daily. The global tea production amount exceeds US$17 billion annually [1], playing an important role in increasing the income of farmers and improving the quality of life in rural areas. Tea yield estimation can provide reliable data support for maximizing income between harvest time and amount, and this support is greatly important in increasing the income of tea farmers [2]. There is currently no efficient method for counting buds and leaves in small-scale tea gardens. Farmers usually count tea buds manually, which is inefficient, time-consuming, and laborious. Therefore, developing an efficient tea bud counting method is necessary. Recently, deep-learning-based method has shown promising prospects for predicting crop yields [3]. However, the counting of tea buds remains hampered by 2 outstanding problems, namely, the low stability of tea bud detection and repeated counting of the same tea bud.

To improve the stability of tea bud detection, detection methods based on traditional image processing algorithm [4–8] and depth learning algorithm [9–12] have been proposed successively. Traditional detection methods mainly based on the machine learning [4] and digital image processing such as texture information [7] and geometric information [8] were employed to identify and detect the target. At present, the tea bud detection method based on convolution neural networks (CNN) has garnered a lot of attention [13]. The YOLO and Faster R-CNN models have got good performance for detecting tea buds, as demonstrated in previous studies [14–17]. These models could gain high accuracy when the background is simple. However, the tea buds are dense and small, and the tea garden environment is unstructured in the actual tea garden. So, the results of these models are not always satisfactory. In addition, attention mechanisms have been verified to improve the detection accuracy of small targets in recent years [18–20]. This approach can be adopted for tea bud detection applications to improve detection accuracy. Therefore, this method could be used for the detection of tea buds to improve its detection accuracy.

When counting in continuous image sequences, it is important to avoid counting the same object multiple times. The counting method based on image sequence collects the target image from multiple perspectives so that the target can be observed more accurately [8,21–22]. For example, Wang et al. [21] counted mangoes in the image sequences. The experimental results showed the prospect that this kind of counting algorithm could be applied in a real field. Among them, Sort [23] and DeepSort [24] algorithms achieve target tracking and counting in continuous image sequences and have been widely used in the counting of pedestrians and vehicles. While the mentioned algorithms perform well in the above fields, they are not completely applicable to the counting of tea buds. This is mainly due to the complex environment of the real tea garden and the small and dense growth of tea buds. In addition, the use of 3-dimensional (3D) technology for counting has also been reported [25]. The problem with this method is that it is too complex and that its hardware cost is too high, which limits its use. Generally speaking, the method of counting objects based on image sequences has a good effect and low cost and has good application prospects. However, because of the complexity of actual tea gardens and the uniqueness of tea buds, the above method may lead to the loss of targets.

**Fig. 1.**
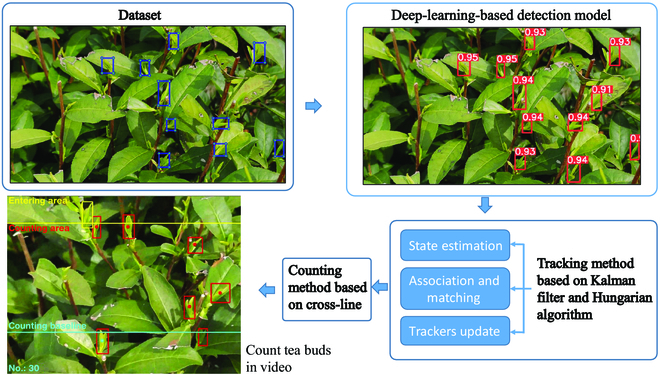
The overview of the proposed method.

Many works have succeeded in detecting and counting crops using a CNN; hence, research on tea bud detection and counting using the same is necessary. Figure 1 presents an overview of the proposed method. First, the image datasets were collected using mobile phones. Then, a tea bud detection model (SE-YOLOv5m) was modified from the object detection algorithm YOLOv5m [26]. One visual attention mechanism called the Squeeze and Excitation Network (SENet) [19,27] is introduced into the CNN to improve the efficiency and accuracy of tea bud detection. Next, the proposed SE-YOLOv5m model and Kalman filter were used in this approach to tracking and counting tea buds. The proposed tracking algorithm was a modified version of DeepSort [24]. Last, the tea bud detection model and counting method were evaluated and tested using test images and videos respectively.

### Related Work

#### Object detection

There are mainly 2 types of object detection algorithms. The first type called single-stage detectors, which could predict the bounding box directly from the input image without a region suggestion step. For example, SSD (Single Shot MultiBox Detector) [28] and YOLO [29] are the typical methods. Sun et al. [30] proposed a light-weight CNN model that can be deployed on mobile devices to detect apple leaf diseases in real time. On the basis of YOLOv5, Qi et al. [10] proposed an improved object detection to recognize of tomato virus diseases. The second type is called 2-stage detectors. It generates region proposals from images first and then extracts features from these regions for classification and positioning. For example, the model of the R-CNN series [31–33] is the typical representative of the method. Yu et al. [34] proposed a strawberry fruit detection method based on Mask R-CNN, which overcame the difficulties of using traditional machine vision algorithms in unstructured environments. A method based on an improved Faster R-CNN using color and depth images was also proposed for the robust detection of small fruits [35]. The single-stage detection methods usually have faster speeds, while the 2-stage detection methods have higher accuracies and can be used for difficult detection tasks.

#### Crop counting methods

The techniques used to achieve crop counting fall into 3 main types. The first category used 3D technology to handle this, which detected and counted objects in a 3D environment [36–38]. This method obtained the position information of the object in 3D space and used the uniqueness of the object in 3D space to realize counting. The second category is the counting-by-regression approach [39]. It trained a regressor to map the local image features into an object density map directly. The third category is the counting-by-detection approach [21,40–47]. This kind of method tracked and counted the target after it was detected. These 3 kinds of methods have their own advantages and disadvantages. The first category of methods is too complex, and its hardware cost is too high, which limits its use. The second method is only suitable for static images and is not suitable for dynamic counting. The third category of methods has a good effect and low cost. However, this kind of method has insufficient adaptability to different objects and environments [40]. Some new tracking strategies and CNN-based detection algorithms can solve this problem. For instance, the counting algorithm based on YOLO and correlation filtering still shows good results in complex environments [21,41].

## Materials and Methods

### Data acquisition and preprocess methods

**Fig. 2.**
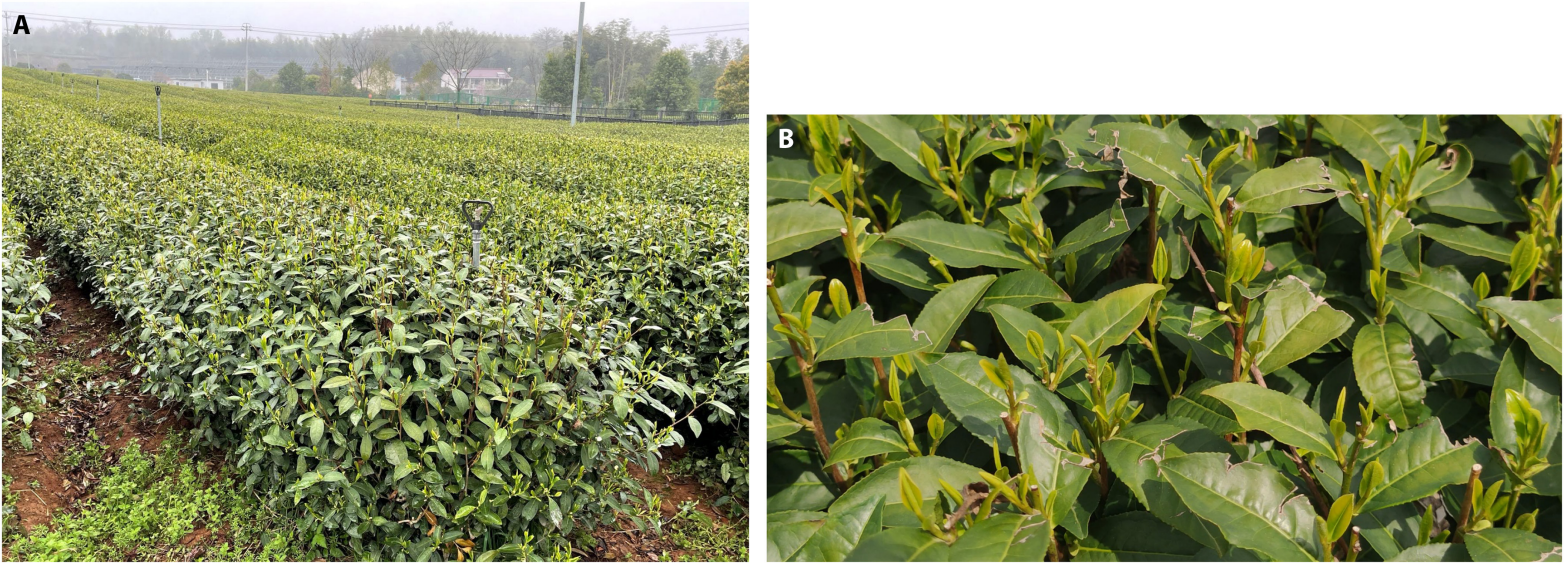
Tea in fields: (A) experimental tea garden and (B) captured image of tea buds.

**Fig. 3.**
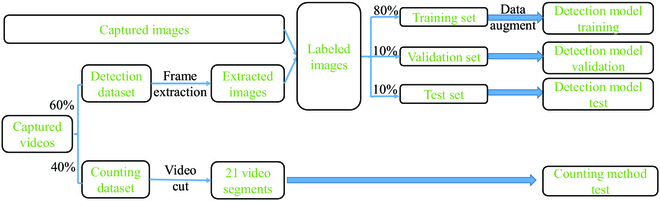
Composition and processing of dataset.

The goal here was to count tea buds using a method that can assist in the tea yield estimation. All images and videos used in this study were collected from the Shengzhou Comprehensive Experimental Base (120.825542E, 29.748715N) of the Tea Research Institute of the Chinese Academy of Agricultural Sciences and shot between 8:30 and 17:00 from 2022 March 15 to 2022 March 31. Figure 2 shows the experimental tea garden and tea buds. Two smartphones [Mate 40 from Huawei (China) and iPhone 8 from Apple (USA)] were used to collect the images and videos of tea leaves under natural illumination. When taking photos and videos, the experimenter slowly walked between the tea ridges at approximately 0.15 m/s. The tilt angles of the smartphones were approximately 45° downward. The shooting distance when capturing images was 30 to 50 cm away from the tea buds. Table 1 shows the overview and description of the dataset used. The composition and processing of dataset is shown in Fig. 3. For each video in the detection dataset, an image is extracted every 10 frames. The overlap between extracted 2 adjacent images accounted for approximately 70% of each image. These images, together with the captured photos, were labeled by LabelImg for making labels of tea buds [48]. Because the sizes of the captured photos and extracted images are not suitable for rapid training, a series of images with the size of 960 × 608 pixels were randomly cropped from them. Finally, a total of 62,235 tea buds were labeled in 4,260 photos. Then, according to the proportion of 8:1:1, all the labeled images were divided into 3 datasets for training, validation, and testing of our tea bud detection models.

**Table 1.** Dataset overview and description.

Items	Values
Number of videos taken	30
Duration of the videos	1 hour in total
Video resolution (pixels)	3,840 × 2,160
Number of images taken	3,000
Number of images selected from the video	1,260
Image resolution (pixels)	3,840 × 2,160, 1,920 × 1,080
The average number of object instances per image	14.6

**Fig. 4.**
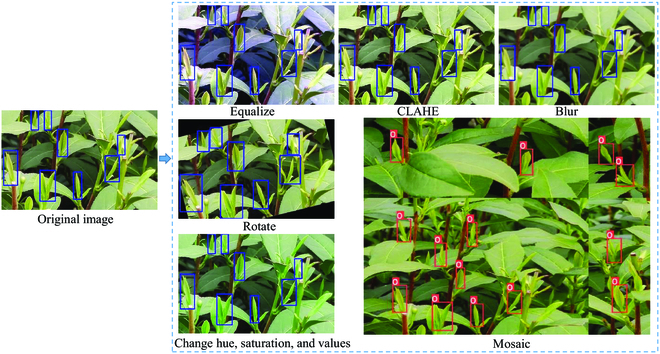
Results of data augmentation. CLAHE, contrast limited adaptive histogram equalization.

During training, a few date augmentation operations (e.g., translate, hue saturation and value change, rotate, flip, blur, contrast limited adaptive histogram equalization, Mosaic [26], and so on) were applied. Some examples of data augmentation operations are shown in Fig. 4.

### Tea bud detection model

**Fig. 5.**
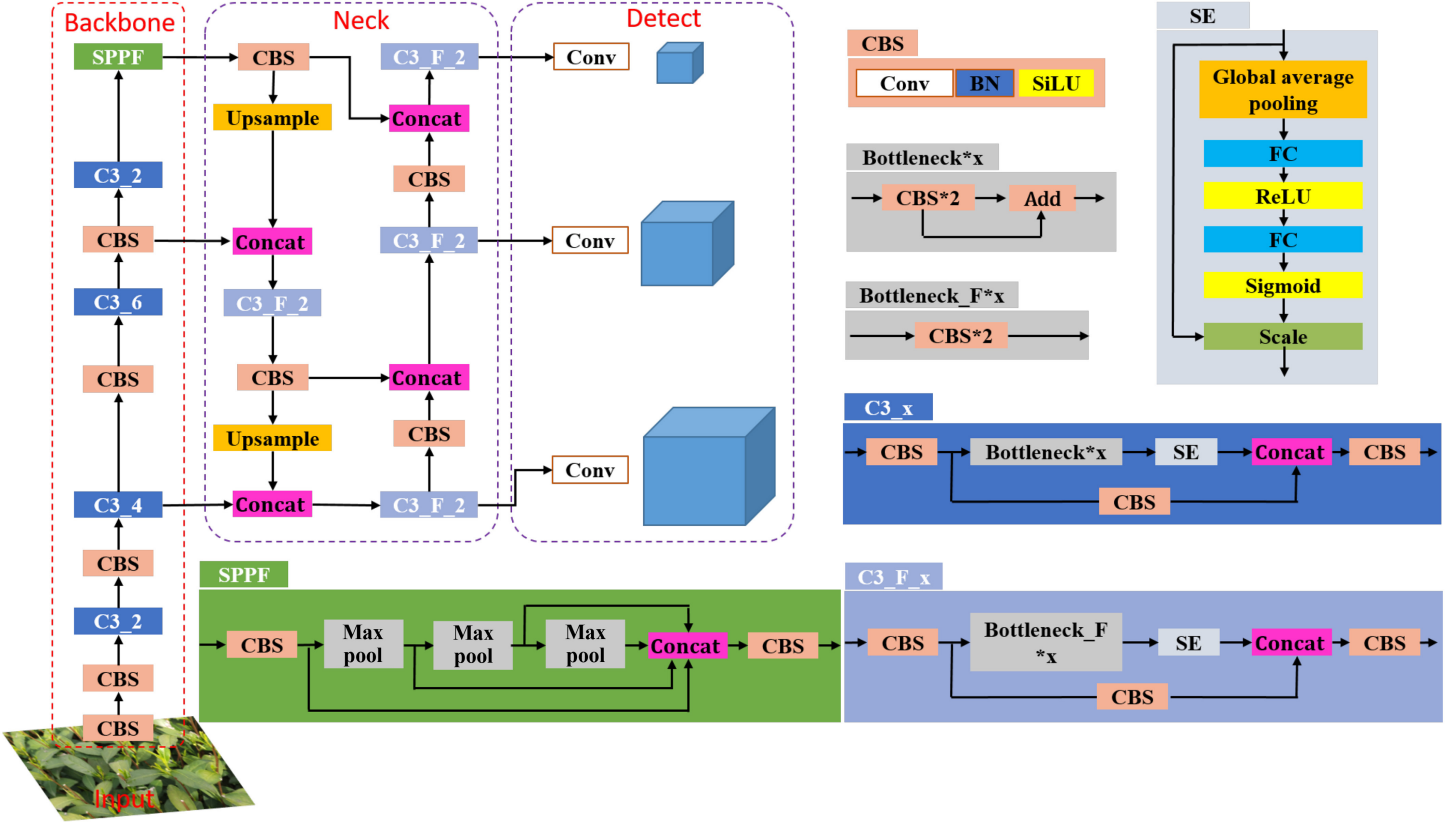
Structure of the proposed model (SE-YOLOv5m). Conv, convolutional layer; BN, batch normalization layer; SiLU, sigmoid liner ReLu; CBS, Conv, BN, and SiLU; C3_x, usage of a CBS structure with X residual modules (ResUnit) (e.g., one residual component is used in the first C3_x; hence, C3_1); SPPF, Spatial Pyramid Pooling - Fast; FC, fully connected layers. The meaning of X in 2 different C3 structures is the same.

YOLOv5 is the fifth generation version of the YOLO series of models and belongs to the category of single-stage object detection models. YOLOv5 could regress the position and category of the target box in the output layer without going through multiple stages. In addition, it has 4 configurations (YOLOv5s, YOLOv5m, YOLOv5l, and YOLOv5x). The depth and width of the model are getting deeper and wider in the order of s, m, l, and x. CSPDarknet53 [49] and the path aggregation network [50] were used in the model backbone and neck, respectively, to make the model lightweight while maintaining accuracy. Related research has shown that incorporating visual attention mechanisms into deep learning models can improve their accuracy in identifying small objects [51]. The SENet [19,27] is a type of convolutional module that improves the representation capability of the network by using weights to filter key features for each convolutional channel. This helps the network to better represent the features and improve its performance. So, it was introduced into the CNN to improve the efficiency and accuracy of tea bud detection. Figure 5 shows the structure of an improved version of SE-YOLOv5m that has been proposed in the current study. This version of SE-YOLOv5m has been designed to enhance the performance of the model. In Fig. 5, the SE module is shown to be used in 2 different C3 modules to improve the feature extraction ability of the model. The SE module consists of 2 main operations: a squeeze operation and an excitation operation. The squeeze operation is performed first, followed by the excitation operation on the global features. The excitation operation calculates weights for each channel and determines the relationship between them. This is done to improve the representation capability of CNN and enhance its performance for detecting tea buds.

### Tea bud counting method based on SE-YOLOv5m

**Fig. 6.**
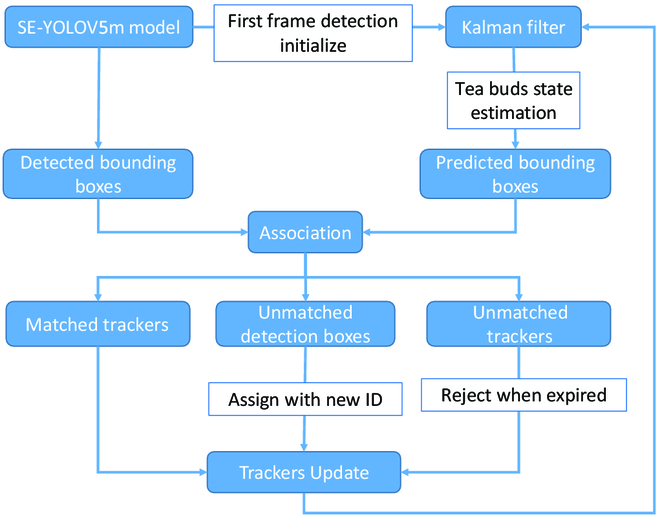
Counting pipeline using the SE-YOLOv5m model and the Kalman-filter-based tracker.

Figure 6 shows the steps involved in the tea buds counting pipeline. To ensure that each tea bud is only counted once, a unique tracking number is assigned to each one. This is done using a tracker based on the Kalman filter [52], which is used to track the tea buds and avoid double counting in continuous image sequences. The process involves 3 steps: (a) estimating the state of the tea buds, (b) matching and associating tea buds between frames, and (c) updating the tracker.

#### Tea bud state estimation

The tea bud tracking problem involves predicting and updating the state variables of a tea bud over time. This is done using a Kalman filter dynamic model, which consists of 2 main steps: prediction and update. In the prediction step, to predict the state variables of the tea bud in the next frame, the state variables of the tea bud in the current frame are used. In the update step, the predicted state variables for the tea bud in the next frame are updated using the observed variables (detected bounding box) in that frame [47]. This process is repeated over time to track the movement of the tea bud. The small position change of the target between video sequences, due to the high frame rate of the camera, allows for the assumption that the camera’s motion is uniform. As a result, the visual detection and tracking system can be treated as being linearly related to the change in time. Therefore, a standard Kalman filter with a constant motion and linear observation model was adopted in this study for the prediction and update of the tea bud state.

#### Association and matching between frames

**Fig. 7.**
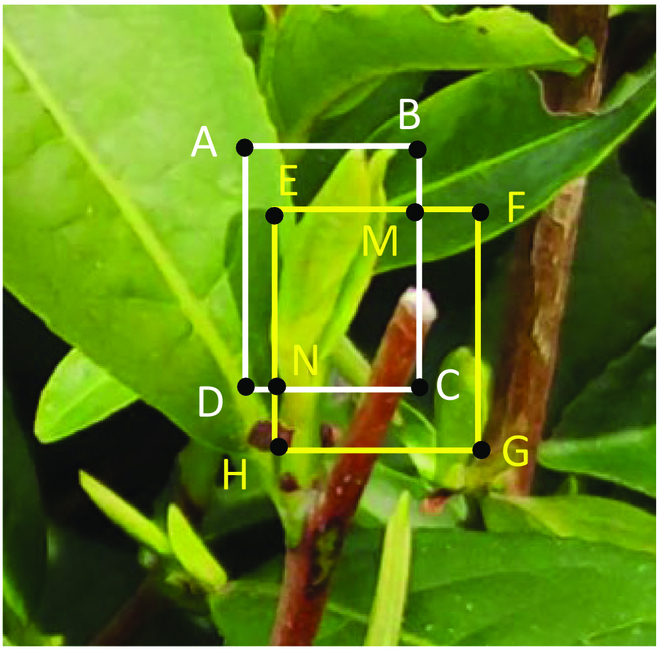
A detection box and a tracker for a tea bud.

In the update step, trackers from the previous frame and detection results from the current frame are used to match with each other. These detection results are considered as ground truth used to update the trackers and the Kalman filter. The Hungarian algorithm [53] is used to create a link between the trackers and the detection results. This helps to update the trackers and the Kalman filter. Figure 7 presents a schematic diagram that demonstrates how the system is able to detect tea buds and track their location using bounding boxes. The diagram shows that the system is able to identify tea buds within an image and then track their movement over time by enclosing them in a bounding box. The white rectangle represents the area of the image that the detector has determined as containing a tea bud, while the yellow rectangle represents the area of the image that the tracker has identified as containing a tea bud and is tracking over time. The IoU (intersection over unit) metric is used to measure the overlap between the bounding boxes predicted by the tracker and the detector (shown in Eq. 1). A higher IoU value indicates a stronger correlation between the 2 bounding boxes.

$$\mathrm{IoU}=\frac{S_{\mathrm{EMCN}}}{S_{\mathrm{ABCD}}+S_{\mathrm{EFGH}}-S_{\mathrm{EMCN}}}$$ (1)

Here, *S*_EMCN_, *S*_ABCD_, and *S*_EFGH_ represent the area of rectangles in Fig. 7, respectively.

#### Tracker update

The process of matching the detected bounding boxes (*D_i_*) and the trackers (*T_i_* − 1) results in 3 categories: trackers that are associated with detection boxes, unmatched detection boxes, and unmatched trackers. Then, in the update process, the trackers associated with detection boxes would be utilized. For detection boxes that did not have a matching tracker, a new tracker is created for each one and added to the set of existing trackers. For trackers that did not match the corresponding detection box, their *V*_1_ value is increased by 1 to reflect that they have experienced one instance of losing their target. It is desirable for each tracker to represent a single tea bud, but the detection model may occasionally fail to detect or incorrectly identify tea buds, resulting in errors in the tracker count and ultimately in the tea bud count. To address these errors, the algorithm uses threshold values *T*_2_ and *T*_3_ to filter out invalid tracker existences and ensure accurate tea bud counts. Unmatched trackers are discarded when they lose the target a certain number of times, as indicated by the *V*_1_ parameter reaching the threshold value *T*_2_. In addition, a valid count for a tea bud is only recorded when the cumulative number of times its associated tracker has existed exceeds *T*_3_.

### Quantitative statistical method based on cross-line counting

**Fig. 8.**
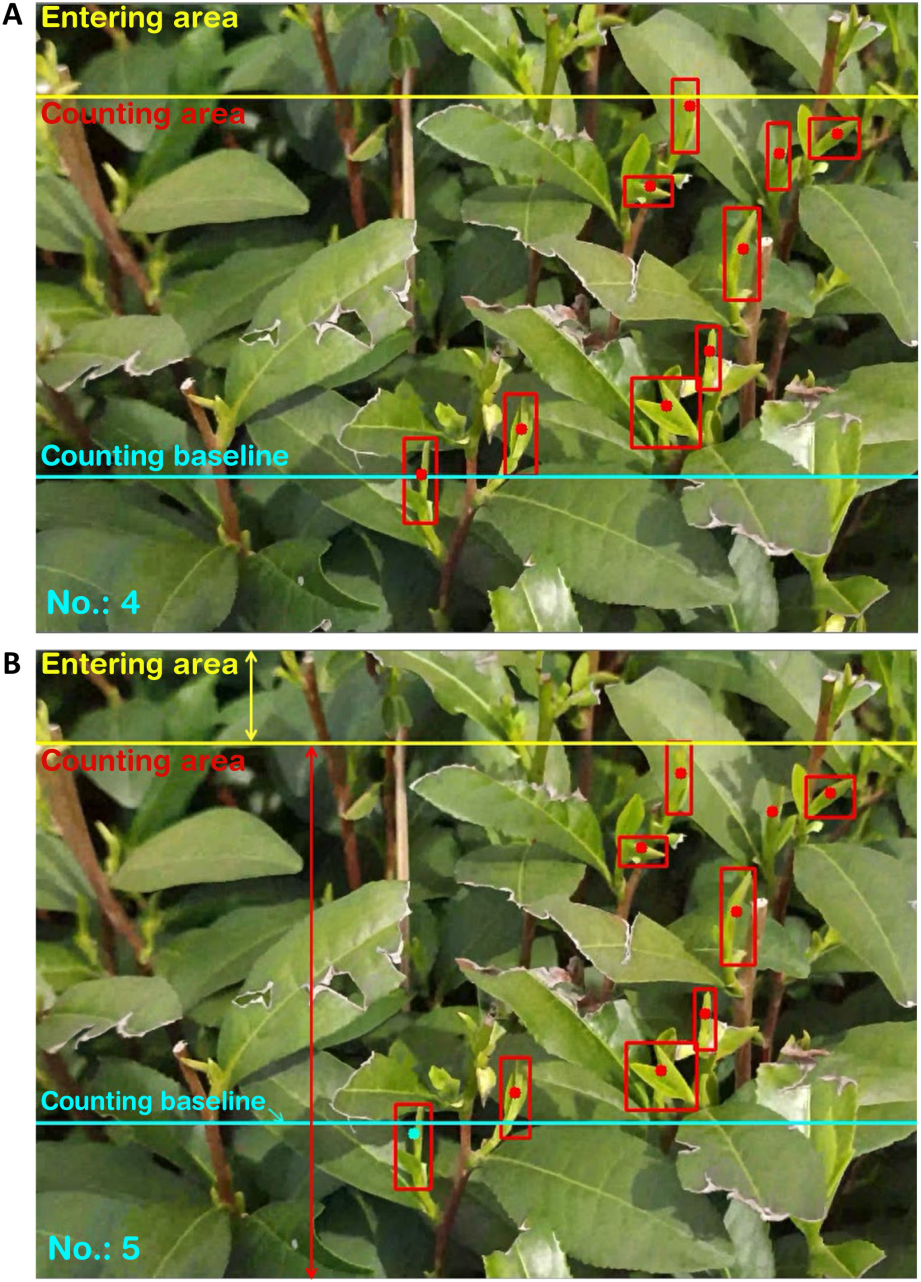
An example of the process of tea buds counting. (A) and (B) depict the counting results on 2 different frames that are separated by a time interval (*i*).

If the tea bud goes undetected by the detection model for a period of time and is subsequently detected again in a later frame, then the tracking ID previously assigned to the tea bud would be discarded, and a new tracking ID would be generated. The view of the plants was prone to distortion when the tea buds appeared at the edge of an image, and this affected the accuracy and reliability of the tracker and the detector. Therefore, to improve the accuracy of the counting process, 2 areas (entering and counting areas) and a line (counting baseline) were defined in the image. The entering and counting areas and the counting baseline were defined at the up, center, and bottom areas of the image in Fig. 8, respectively. The bounding box was not tracked and counted when it was in the entering area. It was tracked only after entering the counting area. The counting baseline, represented by a blue line in the image, was used as a reference for counting tea buds. When a tracked bounding box crossed the counting baseline, it was considered a valid count, and the tracker’s center changed color from red to blue to indicate that it had been counted. This process, shown in Fig. 8, helps to ensure accurate and reliable counting of tea buds.

### Model training and testing

The operating system used for training was Ubuntu 16.04, and the version of Python and Pytorch were 3.7 and 1.8, respectively. The central processing unit and graphics processing unit used were Intel Core I7 6700K (64-GB random access memory) and Nvidia GeForce RTX 3090, respectively. In addition, the CUDA (Compute Unified Device Architecture) and CUDA Deep Neural Network library used were 10.1 and 7.6, respectively. During training, the learning rate, learning rate momentum, batch size, optimizer weight decay factor, and epochs were 0.001, 0.937, 24, 0.0005, and 300, respectively. All other parameters were set to the recommended values provided by the official website. The model weights were initialized using a pretrained weight derived from the Microsoft COCO dataset [54]. In this study, average precision, detection time, and floating point operations (FLOPs) were used to evaluate the performance of the model for detecting tea buds.

## Results and Discussion

### Selection of YOLOv5 model

To select the model suitable for tea bud detection, the 4 configurations of YOLOv5 (s, m, l, and x series) were trained and tested. The comparison results of the models on the test set are shown in Table 2. It can be seen that with the increasing complexity of the YOLOv5 model, its mean average precision (mAP) is increasing, but its detection time and FLOPs are also increasing. Among them, the difference between YOLOv5m and YOLOv5l and between YOLOv5m and YOLOv5x is 0.98% and 1.29%, respectively, while the difference between YOLOv5m and YOLOv5s is 2.1%. Meanwhile, the FLOPs of YOLOv5m is 60.2 and 156.6 G less than those of YOLOv5l and YOLOv5x, respectively. In addition, it is only 34.77 G more than that of YOLOv5s. Thus, the YOLOv5m model was chosen for further development as a tea buds counting method, due to its favorable performance in terms of detection time, FLOPs, and mAP.

**Table 2.** Comparison results of the YOLOv5 (s, m, l, and x series) modes.

Models	mAP (%)	Average detection time (ms)	FLOPs (G)
YOLOv5x	91.35	25.6	205.5
YOLOv5l	91.04	22.4	109.1
YOLOv5m	90.06	20.3	48.9
YOLOv5s	87.96	18.2	16.5

### Evaluation of the proposed tea bud detection model

To test the performance of the proposed tea bud detection model, images in the test dataset were used to evaluate it. In addition, the other state-of-the-art models, namely, Faster R-CNN, SSD, and YOLOv5, were also trained and compared with our method. Faster R-CNN is an improved 2-stage target detection algorithm that has been widely used in tasks such as human posture recognition and target tracking. SSD is a classic one-stage model for fast object detection that balances the detection accuracy and speed well by integrating the regression idea of YOLO and the anchor box mechanism of Faster R-CNN. Table 3 presents the comparison results. As shown in the table, the mAP of SE-YOLOv5m is better than that of SSD model, and its speed is faster than that of SSD model. This shows that the SE-YOLOv5m model is better than the SSD model in these 2 aspects. The mAP of SE-YOLOv5m is close to that of Faster R-CNN, but the detection time of Faster R-CNN is about 9 times that of SE-YOLOv5m. As for SE-YOLOv5m and YOLOv5m, the FLOPs and detection time of them are close, but the difference between their mAPs is 1.82%. This means that SE-YOLOv5m has higher detection accuracy than YOLOv5m. Therefore, considering the accuracy and detection time, our SE-YOLOv5m network was used for counting tea buds in actual tea gardens.

**Table 3.** Proposed tea bud detection model and comparison results.

Models	mAP (%)	Average detection time (ms)	FLOPs (G)
SSD	83.52	44.2	62.79
Faster R-CNN	91.08	180.4	370.41
SE-YOLOv5m	91.88	20.4	49.1
YOLOv5m	90.06	20.3	48.9

### Accuracy evaluation of the model under different image brightness

**Fig. 9.**
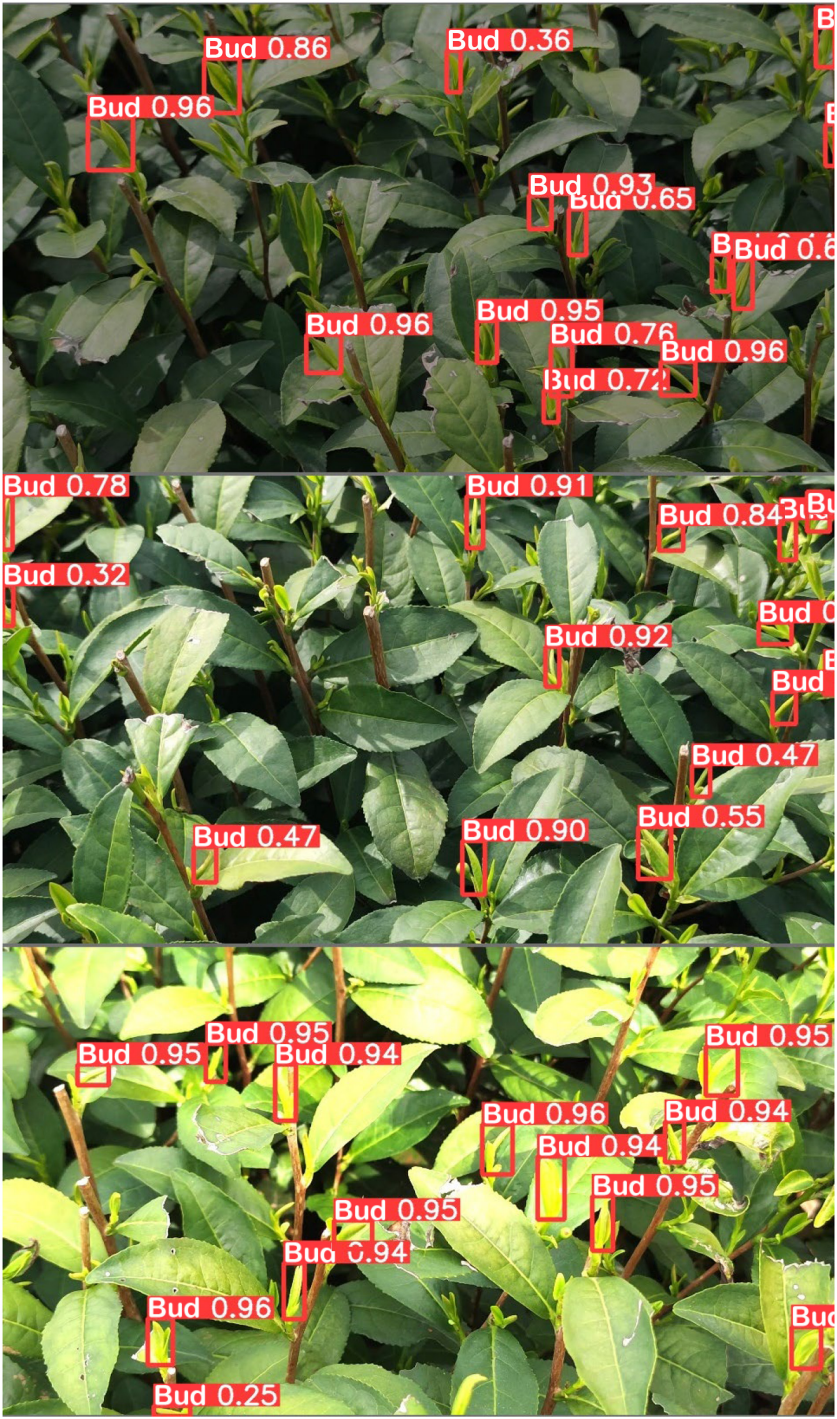
Detection results of SE-YOLOv5m. First to last rows: low-, medium-, and high-brightness images, respectively.

**Fig. 10.**
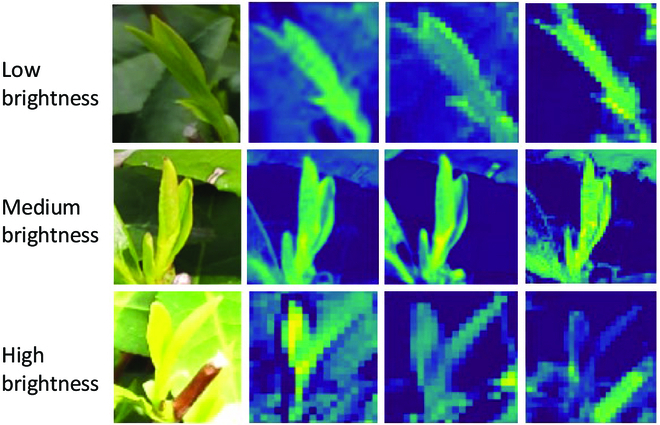
Feature maps of the tea images under different light intensities. Top, middle, and bottom rows: low-, medium-, and high-brightness images, respectively. First to last columns: original images, feature maps of the C3_2 module, feature maps of the C3_4 module, and feature maps of the C3_6 module in the SE-YOLOv5m model, respectively.

The detection and counting accuracies of the method may be affected by the uneven brightness of the tea images collected at different times under natural light. Therefore, the above test set was divided according to different image brightness. Brightness is represented by the average gray value of the corresponding gray image. According to the image brightness and darkness, the tea images were divided into 3 intervals according to the average gray value, that is, [30, 90), [90, 170), and [170, 230], which represent the low- (A), and medium- (B), and high- (C) brightness datasets, respectively. The tested model was the SE-YOLOv5m model trained above. Table 4 shows the detection results. Figure 9 depicts the test sample results under different brightness. In the table, the precision and the recall rate were both approximately 91% when under low and medium brightness. This indicated that medium- and low-brightness images had a slight influence on model detection. The bud detection precision for high-brightness images was 89.22%, but the detection recall rate was only 72.32%, implying a certain degree of missed detection for the high-brightness images. Figure 10 illustrates the convolution feature maps of the tea images under different light intensities to further analyze the reasons for the results. The tea bud features are clear and stable in the first 3 layers of the convolution feature map when the tea images are under medium and low brightness. In addition, the old leaf features weaken as the number of network layers increases. The difference between the features of the old leaves and the buds, however, was not as apparent as that under medium and low brightness when the tea images were under high brightness. The effective features of high-brightness images become weaker as the number of network layers increases. Therefore, it was difficult to learn effective features under high-brightness images. The model’s detection performance would degrade under this condition.

**Table 4.** Proposed tea bud detection model and comparison results under different weed population densities.

Dataset	Precision (%)	Recall (%)	Number of images
Dataset A	91.96	89.69	140
Dataset B	92.66	91.46	156
Dataset C	89.22	72.32	130

### Evaluation of the counting method

**Fig. 11.**
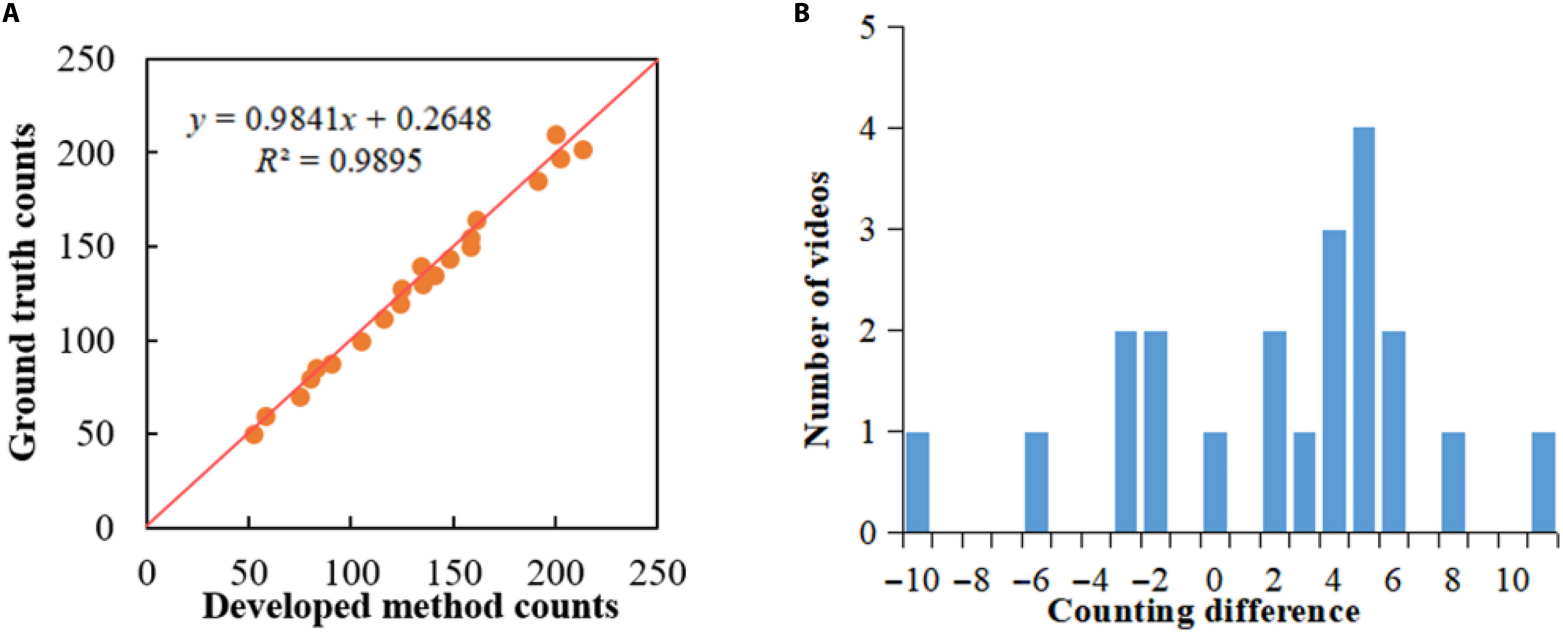
Results of the manual and algorithmic count regression analyses.

To test the proposed counting method, it was evaluated on 21 video segments that were cut from the counting dataset. The length of the tea ridge contained in each video segment is about 1.5 m. The number of tea buds in each video clip was counted by 3 people manually, and the average of 3 values was taken as the final number. Figure 11 shows the results of the manual and algorithmic count regression analyses. In Fig. 11A, the counting algorithm results for the tea buds were highly similar to those of manual counting (*R*^2^ = 0.98). For all the test videos, the absolute count error of 85.7% of the test results was less than or equal to 6, and that of 14.3% of the test results was greater than 6. Figure 11B also illustrates that 90.4% of the testing videos have an absolute count error of less than 8, which is considered an acceptable counting accuracy. This expresses the potential possibility that the calculated results of this method can be directly used for tea bud counting.

## Conclusion

The following results were obtained in this study:

1. A tea bud detection model (SE-YOLOv5m) was modified from the object detection algorithm YOLOv5m. The SENet is introduced into the CNN to make the efficient and accurate detection of tea buds in a tea garden. The mAP on the test dataset is 91.88% (IoU, 0.5), which shows its effectiveness for tea bud detection. The evaluation results under different brightness images showed clear and stable tea bud features of the tea tree images in the medium- and low-brightness images. By contrast, learning effective features was difficult in high-brightness images.
2. A tea bud counting method that is suitable for a tea garden was proposed herein. The proposed SE-YOLOv5m model and Kalman filter were used in this approach. The counting experiment results showed that the tea bud counting algorithm was highly similar to that of manual counting (*R*^2^ = 0.98); hence, the developed method could accurately and effectively count tea buds. The database and method used in this study would provide data and technical support for others to further study tea bud detection and counting methods.

## Data Availability

The image dataset used to support the findings of this study is available from the corresponding authors upon request.
